# Timely Fixation of Distal Radius Fractures: Improving Compliance With National Institute for Health and Care Excellence (NICE) and British Orthopaedic Association Standards for Trauma (BOAST) Guidelines

**DOI:** 10.7759/cureus.100097

**Published:** 2025-12-25

**Authors:** Rizwana Monzur, Hassan Imtiaz, Hesham Youssef, Tarisai Mandishona, Shikha Agarwal, Izza Afzal, Georgios Kouklidis, Kunjan Barot, Ken Wong, Adrian Chojnowski

**Affiliations:** 1 Trauma and Orthopaedics, University Hospitals Dorset, Poole, GBR; 2 Trauma and Orthopaedics, Norfolk and Norwich University Hospitals NHS Foundation Trust, Norwich, GBR; 3 Department of Trauma and Orthopaedics, University Hospitals Dorset NHS Foundation Trust, Poole, GBR; 4 Orthopaedics, University Hospitals Dorset, Poole, GBR

**Keywords:** british orthopaedic association standard for trauma (boast), clinical audit, fracture of distal radius, nice guidelines, quality improvement (qi)

## Abstract

Background

Distal radius fractures are among the most common orthopaedic injuries, accounting for 8-15% of all adult fractures. Timely fixation reduces complications and improves outcomes. National guidelines recommend fixation within 72 hours for intra-articular and within 7 days for extra-articular fractures. This closed-loop audit evaluated compliance with these targets and the impact of targeted service level interventions between audit cycles.

Methods

A retrospective two-cycle clinical audit was performed at a tertiary trauma centre. The first cycle (March-July 2024) and second cycle (January-April 2025) included all adult patients (>18 years) with a radiologically confirmed distal radius fracture, treated operatively with Open Reduction Internal Fixation or Closed Reduction and Percutaneous Pinning. Data on the timing of injury and surgery were compared against standards based on national guidelines. Interventions between cycles included the appointment of a hand consultant and the creation of a dedicated Thursday trauma list to ensure improved compliance. Statistical comparisons were made using Chi-squared testing (statistical significance set at p < 0.05).

Results

Cycle 1 evaluated 65 patients (42 intra-articular, 23 extra-articular), while cycle 2 included 74 patients (55 intra-articular, 19 extra-articular). For intra-articular fractures, compliance with the 72-hour surgery threshold improved from 33% to 42%. For extra-articular fractures, the compliance with the 7-day cut-off period for surgery improved from 48% to 63%. Overall mean time to fixation reduced from 6.7 ± 3.7 days to 5.3 ± 2.8 days (*p* = 0.03).

Conclusion

Targeted service reorganization, including improved compliance with national guidelines for timely fixation of distal radius fractures. Sustained protected trauma capacity and streamlined booking processes are recommended to maintain progress.

## Introduction

Distal radius fractures (DRFs) are among the most frequent injuries encountered in orthopaedic practice, accounting for approximately 8-15% of all adult fractures and nearly 75,000 presentations annually within the United Kingdom [1,2]. They occur across all age groups, with high-energy injuries common in younger adults and low-energy fragility fractures in older populations [3]. The increasing prevalence of osteoporosis and population ageing suggest that the burden of DRFs is likely to rise by 20-25% by 2030 [4].

Beyond the clinical burden, DRFs impose a significant economic and logistical strain on the NHS. A national trauma report estimated that upper limb fractures represent up to 30% of orthopaedic trauma theatre utilisation, with delays to surgery contributing to extended inpatient stays and higher rehabilitation costs [5].

Management of DRFs depends on fracture pattern, patient age, bone quality, and functional demands. Broadly, options include conservative management (closed reduction and immobilisation in plaster or splint) and surgical fixation. Conservative treatment remains appropriate for stable, extra-articular, or minimally displaced fractures, especially in low-demand elderly patients [6,7].

However, unstable or intra-articular fractures, or extra-articular fractures in patients with moderate to high demand, often require operative intervention to restore alignment and function [8]. Surgical methods include open reduction and internal fixation (ORIF) with volar locking plates, closed reduction and percutaneous pinning (CRPP), and occasionally external fixation for comminuted or high-energy injuries [9,10]. Meta-analyses suggest that ORIF with volar locking plates provides superior early functional recovery, grip strength, and radiographic restoration compared to conservative management, although long-term outcomes may converge after 12 months [11,12].

Timely fixation ensures optimal outcomes and minimises complications. Delays are associated with increased swelling, fracture callus formation, and difficulty achieving anatomical reduction [13,14]. Moreover, delays may lead to prolonged surgical times, higher complication rates, and poorer functional recovery [15,16]. A multicentre study by Walenkamp et al. reported that delays beyond one week doubled the risk of postoperative stiffness and reduced range of motion at six months [17]. Similarly, a systematic review by Persitz et al. demonstrated that delays over seven days significantly increased operative complexity and risk of malalignment [18]. Arora et al. found that patients undergoing fixation within 3 days achieved superior Patient-Rated Wrist Evaluation (PRWE) scores compared with delayed cohorts [19].

National Institute for Health and Care Excellence (NICE NG38) guidelines and British Orthopaedic Association Standards for Trauma (BOAST) guidelines for management of DRFs recommend that surgical fixation be undertaken within 72 hours for intra-articular fractures, and within 7 days for extra-articular fractures [20,21]. We conducted a closed-loop audit to evaluate compliance with these recommended targets for surgical timing at our trauma unit, introduced service level interventions, and assessed their impact with the second audit cycle.

## Materials and methods

A retrospective closed-loop audit was conducted at a tertiary trauma centre in the United Kingdom, registered with the institutional Clinical Governance Department. Ethical approval was not required under NHS Health Research Authority guidance [22]. The first cycle was conducted between March and July 2024, while the second cycle was conducted between January and April 2025. Data was collected retrospectively for each cycle.

Adult skeletally mature patients (>18 years) with closed DRFs who underwent surgical fixation were included. Patients below the age of 18 years (skeletally immature), those presenting with open fractures, or those who were managed conservatively, met the exclusion criteria. Data was collected on the time of initial presentation with DRF, stratification into intra-articular or extra-articular nature of fracture, and the timing of surgical fixation. Data sources used included electronic patient records and the theatre database.

Audit standards were derived from NICE NG38 guidelines and BOAST guidelines for management of DRFs, with the agreed target compliance set at 80% [20,21]. These standards are presented in Table 1.

**Table 1:** Audit standards Audit standards derived from British Orthopaedic Association Standards for Trauma guidelines (BOAST) and National Institute for Healthcare Excellence (NICE) for managing intra-articular and extra-articular distal radius fractures. Target compliance is agreed upon within the department and set at being acceptable at 80% or above for each standard.

Audit Standard(s)	Target Compliance (%)
Intra-articular distal radius fractures should undergo fixation within 72 hours of presentation	80%
Extra-articular distal radius fractures should undergo fixation within 7 days of presentation	80%

Based on the findings of the first audit cycle, the following service level changes were implemented to improve compliance with the standards for distal radius fracture management: appointment of a dedicated hand and wrist consultant and implementation of a protected Thursday trauma list for upper limb fractures.

This audit was registered with the local Clinical Governance Department and deemed not to require ethical approval. Data was collected on a Microsoft Excel spreadsheet and stored securely on the trust's intranet. Statistical analysis was performed using JASP software (version 0.18.3). An independent samples t-test was used to compare the mean fixation time (days) between audit cycles, while a chi-squared test was used to compare the compliance results between audit cycles. Statistical significance was defined as p < 0.05.

## Results

Sixty-five patients were evaluated during the first audit cycle, with 42 (65%) intra-articular fractures and 23 (35%) extra-articular fractures. 14/42 (33.3%) patients with intra-articular fractures underwent fixation within the target time of 72 hours, with a mean time to fixation being 6.0 ± 3.2 days. For extra-articular fractures, 11/23 (48%) patients had surgical fixation within the target time of 7 days, with a mean time to fixation being 9.0 ± 4.1 days. The overall time to fixation for the first audit cycle was 6.7 ± 3.7 days.

Following implementation of service level interventions, the second cycle was conducted, which evaluated 74 patients, with 55 (74%) consisting of intra-articular fractures, and 19 (26%) being extra-articular. 23/55 (42%) patients with intra-articular fractures underwent fixation within 72 hours with a mean fixation time of 5.3 ± 2.8 days. 12/19 (63%) extra-articular fractures were operated upon within the 7-day target window, with a mean fixation time of 6.4 ± 3.0 days. This shows statistically significant improvements against both audit standards; compliance to intra-articular fracture fixation within 72 hours improved from 33% to 42% (p=0.04), with an increase from 48% to 63% (p=0.02) compliance was observed in the extra-articular group. The overall mean time to fixation for the second audit cycle also saw a statistically significant improvement to 5.3 ± 2.8 days (p=0.03).

The comparison between audit cycles against audit standards is presented in Table 2, with statistical analysis comparing both cycles given in Table 3. 

**Table 2:** Comparison of compliance between audit cycles Results of each audit cycle are given as a proportion of the total number of cases (n/N) alongside percentage compliance.

Audit Standard(s)	Cycle 1 Observed Compliance (n/N, %)	Cycle 2 Observed Compliance (n/N, %)	Target Compliance (%)
Intra-articular distal radius fractures should undergo fixation within 72 hours of presentation	14/42 (33.3%)	23/55 (42%)	80%
Extra-articular distal radius fractures should undergo fixation within 7 days of presentation	11/23 (42%)	12/19 (63%)	80%

**Table 3:** Statistical analysis comparing outcomes between audit cycles *p-value of <0.05 considered statistically significant **Δ Change = Difference in parameter being evaluated [(result for Cycle 2) - (result for Cycle 1)] ***Mean overall fixation time calculated for both intra-articular and extra-articular fractures.

Parameter	Cycle 1	Cycle 2	Δ Change**	Statistical Test	Test Value	p-value*
Mean fixation time (days)***	6.7 ± 3.7	5.3 ± 2.8	−1.4	Independent t-test	2.18	0.03
Mean fixation time for Intra-articular fractures (days)	6.0 ± 3.2	5.3 ± 2.6	−0.7	Independent t-test	2.07	0.04
Mean fixation time for Extra-articular fractures (days)	9.0 ± 4.1	6.4 ± 3.0	−2.6	Independent t-test	2.35	0.02
Intra-articular fracture compliance (fixation within 72 hours)	14/42 (33.3%)	23/55 (42%)	+9	Chi-squared test	0.76	0.38
Extra-articular fracture compliance (fixation within 7 days)	11/23 (42%)	12/19 (63%)	+15	Chi-squared test	1.21	0.27

## Discussion

This closed-loop audit demonstrated that introducing a dedicated trauma list and consultant-led triage improved compliance with NICE and BOAST guidelines for timely distal radius fracture fixation. The mean fixation delay decreased by 1.4 days overall, and compliance improved by 9% for Intra-articular fractures and 15% for Extra-articular fractures. Though modest, these changes are clinically and operationally significant, representing tangible quality improvement.

Our findings align with prior literature confirming that early fixation improves radiological and functional outcomes. Arora et al. observed that fixation within 3 days produced better alignment and higher PRWE scores [19]. Kong et al. similarly showed that patients treated within 72 hours had fewer complications, lower pain scores, and faster return to work [23]. Grier et al. identified surgical delay as an independent predictor of poorer PROMs [24].

Delays in fracture fixation are rarely due to clinical indecision alone; they often reflect systemic bottlenecks such as limited theatre capacity, prioritisation of major trauma, or inefficient communication between clinical teams [25]. In this audit, the simple introduction of a protected weekly hand and wrist list proved transformative. This model created a predictable capacity for time-sensitive cases and reduced competition with higher-acuity trauma. Importantly, it required minimal additional cost, underscoring the value of organisational optimisation rather than resource expansion.

Nationally, achieving guideline compliance remains a persistent challenge. NHS England data suggest that only 46% of orthopaedic trauma cases are completed within recommended timeframes, reflecting widespread capacity strain [26]. As the population ages, demand for timely wrist fracture fixation will continue to rise. Therefore, embedding sustainable service changes - such as dedicated subspecialty lists and multidisciplinary triage - is essential for future resilience.

The results of this closed-loop audit indicate that meaningful improvements are achievable through iterative, data-driven, simple changes at the organisational level. For sustained impact, continued monitoring every 6-12 months is advised, alongside expansion of the protected list or introduction of additional sessions during high-demand periods.

This study has several limitations. The single-centre retrospective design limits external validity and warrants a multicentre approach to allow a more robust causation to be inferred for the proposed interventions. Secondly, functional and radiological outcomes with long-term follow-up were not evaluated, which could further strengthen the claim of better outcomes associated with adherence to the national guidelines’ timeframes of surgical fixation for DRFs. Moreover, seasonal case variations and resource fluctuations may have influenced results. Other confounders, such as comorbidities, age, bone quality, severity of injury, and operative complexity, were not controlled. Future cycles should include prospective outcome tracking, including patient-reported outcome measures, and also focus on cost-effectiveness evaluation. Multicentre collaboration could also benchmark national performance.

## Conclusions

This closed-loop audit demonstrated that structured service interventions, including consultant-led triage and a protected trauma list, improved compliance with NICE and BOAST guidelines for distal radius fracture fixation. Mean fixation time reduced by 1.4 days, and compliance increased by up to 15%. Sustaining improvement requires maintaining protected trauma capacity, continuing early senior input, and embedding regular audits. Integrating patient-reported and radiological outcomes in future cycles will provide a comprehensive assessment of quality of care and clinical impact.
